# Pterostilbene, a Methoxylated Resveratrol Derivative, Efficiently Eradicates Planktonic, Biofilm, and Intracellular MRSA by Topical Application

**DOI:** 10.3389/fmicb.2017.01103

**Published:** 2017-06-13

**Authors:** Shih-Chun Yang, Chih-Hua Tseng, Pei-Wen Wang, Po-Liang Lu, Yi-Han Weng, Feng-Lin Yen, Jia-You Fang

**Affiliations:** ^1^Pharmaceutics Laboratory, Graduate Institute of Natural Products, Chang Gung UniversityTaoyuan, Taiwan; ^2^School of Pharmacy, College of Pharmacy, Kaohsiung Medical UniversityKaohsiung, Taiwan; ^3^Research Center for Natural Products and Drug Development, Kaohsiung Medical UniversityKaohsiung, Taiwan; ^4^Center for Infectious Disease and Cancer Research, Kaohsiung Medical UniversityKaohsiung, Taiwan; ^5^Department of Fragrance and Cosmetic Science, College of Pharmacy, Kaohsiung Medical UniversityKaohsiung, Taiwan; ^6^Department of Medical Research, China Medical University Hospital, China Medical UniversityTaichung, Taiwan; ^7^Department of Internal Medicine, Kaohsiung Medical University HospitalKaohsiung, Taiwan; ^8^College of Medicine, Kaohsiung Medical UniversityKaohsiung, Taiwan; ^9^Institute of Biomedical Sciences, National Sun Yat-Sen UniversityKaohsiung, Taiwan; ^10^Research Center for Food and Cosmetic Safety and Research Center for Chinese Herbal Medicine, Chang Gung University of Science and TechnologyTaoyuan, Taiwan; ^11^Department of Anesthesiology, Chang Gung Memorial HospitalTaoyuan, Taiwan

**Keywords:** pterostilbene, resveratrol, MRSA, skin infection, biofilm, proteomics

## Abstract

Pterostilbene is a methoxylated derivative of resveratrol originated from natural sources. We investigated the antibacterial activity of pterostilbene against drug-resistant *Staphylococcus aureus* and the feasibility of using it to treat cutaneous bacteria. The antimicrobial effect was evaluated using an *in vitro* culture model and an *in vivo* mouse model of cutaneous infection. The minimum inhibitory concentration (MIC) assay demonstrated a superior biocidal activity of pterostilbene compared to resveratrol (8~16-fold) against methicillin-resistant *S. aureus* (MRSA) and clinically isolated vancomycin-intermediate *S. aureus* (VISA). Pterostilbene was found to reduce MRSA biofilm thickness from 18 to 10 μm as detected by confocal microscopy. Pterostilbene showed minimal toxicity to THP-1 cells and was readily engulfed by the macrophages, facilitating the eradication of intracellular MRSA. Pterostilbene exhibited increased skin absorption over resveratrol by 6-fold. Topical pterostilbene application improved the abscess formation produced by MRSA by reducing the bacterial burden and ameliorating the skin architecture. The potent anti-MRSA capability of pterostilbene was related to bacterial membrane leakage, chaperone protein downregulation, and ribosomal protein upregulation. This mechanism of action was different from that of resveratrol according to proteomic analysis and molecular docking. Pterostilbene has the potential to serve as a novel class of topically applied agents for treating MRSA infection in the skin while demonstrating less toxicity to mammalian cells.

## Introduction

*Staphylococcus aureus* (*S. aureus*) is the main cause of bacterial infection in community settings and hospitals. The mortality produced by *S. aureus* is higher than the sum of viral hepatitis, human immunodeficiency virus, tuberculosis, and influenza in the United States (Hoyert and Xu, 2012). Conventional antibiotics have the ability to fight against *S. aureus* infection. Nevertheless, the multi-drug-resistant *S. aureus* strains such as methicillin-resistant *S. aureus* (MRSA) are an increasing health threat and economic burden. It is reported that there were 23,000 deaths due to MRSA in the United States in 2014 (Barman et al., 2016). *S. aureus* is frequently found in the skin where it may cause cellulitis, folliculitis, and abscess (Water et al., 2015). The management of cutaneous infection has been complicated by the emergence of MRSA. Moreover, the infections associated with biofilm and intracellular MRSA are difficult to cure because of their inherent resistance to antimicrobial drugs and host cells. Biofilm is the bacterial community enclosed by the self-secreted matrix and extracellular polymeric substance forming the barrier that prevents the penetration of antibiotics (Chung and Toh, 2014). The microorganisms become recalcitrant through intracellular persistence in mammalian cells. This urges the development of novel antibacterial agents to overcome the biocidal resistance.

**Figure 1:**
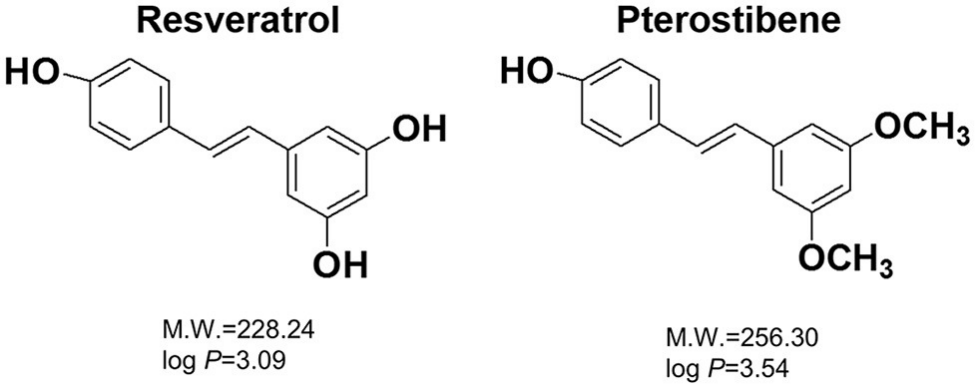
The chemical structures, molecular weights (M.W.), and partition coefficients (log *P*) of resveratrol and pterostilbene. The log *P* was calculated by Discovery Studio® (Accelrys, San Diego, CA, USA).

Resveratrol is a natural compound derived from grapes, peanuts, cranberries, and other botanical sources (Figure 1). In addition to its wide bioactivities, resveratrol can inhibit the growth of some pathogenic bacteria and fungi (Chan, 2002; Paulo et al., 2010; Park et al., 2016). Pterostilbene is a methoxylated form of resveratrol that primarily exists in blueberries (Figure 1). It exhibits chemopreventive, anti-inflammatory, anti-atherosclerotic, and neuroprotective activities (Kosuru et al., 2016). Recently, clinical trials have been conducted to assess the prevention of cardiac diseases by pterostilbene (Riche et al., 2014). It has been reported that pterostilbene can assist anti-MRSA activity of oxacillin and may show potent eradication against *Candida albicans* biofilm (Li et al., 2014; Ishak et al., 2016). Some reports have been published to approve the possible candidacy of pterostilbene as an antibacterial agent; however, information about *in vivo* efficacy and the mechanism of action is still lacking. The aim of this study was to evaluate pterostilbene for biocidal activity against planktonic, biofilm, and intracellular bacteria as well as for its *in vivo* capability in skin-infection inhibition.

A panel of MRSA, *Pseudomonas aeruginosa* (*P. aeruginosa*), and drug-resistant clinical isolates was selected to test the antimicrobial activity of pterostilbene and resveratrol for comparison. The cutaneous targeting favors more drug retention in the skin for efficient bacterial eradication. We compared the cutaneous absorption of pterostilbene and resveratrol to evaluate the targeting ability. Whether the skin was tolerant to topically applied pterostilbene was investigated *in vitro* and *in vivo*. Finally, we explored the mechanisms of antibacterial activity of pterostilbene by proteomic analysis and molecular docking.

## Materials and methods

### Bacterial strains and culture conditions

Table 1 summarizes the strains employed in this report. We used the Gram-positive (MRSA, ATCC 33591) and Gram-negative (*P. aeruginosa*) bacteria. Four drug-resistant clinical isolates were used, two of which were MRSA (KM-1 and KM-2). The other two isolates were vancomycin-intermediate *S. aureus* (VISA, KV-1, and KV-5). All clinical strains were provided by Kaohsiung Medical University Hospital. The bacteria were grown in trypticase soy broth (TSB) at 37°C until achieving OD_600_ = 3.0 under aerobic conditions.

**Table 1:** The MIC and MBC of MRSA, VISA, and *P. aeruginosa* after treatment of resveratrol and pterostilbene.

**Bacteria**	**MIC (mM)**		**MBC (mM)**	
	**Resveratrol**	**Pterostilbene**	**Resveratrol**	**Pterostilbene**
MRSA (ATCC 33591)	1.25	0.16~0.63	5.00~10.00	0.16~0.63
MRSA (KM-1)	1.25	0.078	5.00~10.00	0.078~0.156
MRSA (KM-2)	1.25	0.078	5.00~10.00	0.078
VISA (KV-1)	1.25	0.078	5.00~10.00	0.078~0.156
VISA (KV-5)	1.25	0.078	5.00~10.00	0.078~0.156
*P. aeruginosa*	5.00	5.00	10.00	5.00~10.00

*KM-1, KM-2, KV-1, and KV-2 are the clinical isolates of bacteria*.

*Each value represents the mean ± S.D. (n = 3)*.

### Minimum inhibitory concentration (MIC) and minimum bactericidal concentration (MBC)

The antibacterial activity of resveratrol and pterostilbene (Sigma-Aldrich, St. Louis, MO, USA). was evaluated by MIC and MBC determination. A 2-fold broth-dilution method was utilized to assess MIC. The bacterial population was exposed to several dilutions of compounds ranging from 0.018 to 10 mM and incubated at 37°C for 16 h. An ELISA reader was used to detect MIC at 595 nm. MIC was measured as the highest dilution revealing no bacterial growth. For MBC assay, the bacteria were diluted in PBS and positioned on plates. The compounds with different dilutions were incubated with the microorganisms for 16 h. The colony-forming unit (CFU) was counted. The highest dilution, which resulted in a 99.9% reduction of cell numbers, was recognized as MBC.

### Bacterial survival detected by confocal microscopy

The viability of MRSA after resveratrol or pterostilbene treatment at 1.25 mM was examined using SYTO9 reagent (Molecular Probes, Eugene, OR, USA). The bacterial pellet was obtained by centrifugation at 12,000 rpm for 3 min. The pellet was resuspended in culture medium (1 ml) with the compounds. After treatment at 37°C for 4 h, the samples were stained with SYTO9 and incubated for 15 min. The samples were visualized under a confocal microscope (Leica TCS SP8, Wetzlar, Germany).

### Disk diffusion assay

The assay was performed by plating MRSA (OD_600_ = 0.8) on the agar plate. The 6 mm-diameter disk was put on the agar medium, and the compounds (1.25 mM) with a volume of 10 μl were pipetted into the disk. The plate was incubated at 37°C for 12 h. Subsequently, the diameter of the inhibition zone was measured.

### Total protein amount

MRSA was grown in TSB to OD_600_ = 1.0 and then treated with the compounds at 2 mM at 37°C for 2 h. After centrifugation, the pellet was resuspended with water (0.5 ml). Following sonication for 20 min, MRSA was centrifuged at 4°C and 10,000 rpm for 15 min. The total protein amount of MRSA was estimated using a Bio-Rad protein assay kit (Hercules, CA, USA) in ELISA at 595 nm.

### Transmission electron microscopy (TEM)

MRSA at OD_600_ = 1.0 was treated with pterostilbene (0.5 mM) in TSB at 37°C for 4 h. The bacteria were fixed with 4% glutaldehyde, followed by 1% osmium tetroxide fixation for 2 h. After dehydration in an ascending series of ethanol, the sample was embedded in Spurr's resin. The bacteria sample on the carbon grid was then observed under TEM (JEOL JEM-1200Ex, Tokyo, Japan).

### Biofilm assay

The MRSA biofilm was grown in a Cellview® dish by incubating the bacteria (OD_600_ = 0.1) in TSB containing 1% glucose at 37°C for 24 h. The biofilm was then treated with pterostilbene at a dose of 0.5 mM for 24 h. The biofilm was marked by a Live/Dead Baclight® kit (Molecular Probes) for 15 min. The biofilm was gently rinsed with PBS. The 3D biofilm structure and the biofilm thickness was determined by confocal microscopy.

### Intracellular MRSA killing

The macrophages differentiated from THP-1 monocytes by phorbol myristate acetate (0.1 μM) were used as the host cells to evaluate the activity of pterostilbene toward intracellular bacteria. THP-1 were infected by MRSA at different cell numbers (2 × 10^5^, 2 × 10^6^, 5 × 10^6^, and 1 × 10^7^ cells/ml) for 20 min. After being washed with PBS, the cells were incubated with pterostilbene at 0.1 mM. After a 4-h period, the samples were rinsed with PBS. Triton X-100 (1%) was incorporated into the medium for cell lysis. The resultant solution was cultured on the agar plate for 20 h to count CFU.

### Animals

Female nude mice (8 weeks old) were obtained from National Laboratory Animal Center (Taipei, Taiwan). The experiments were performed in strict accordance with the recommendations set forth in the Guidelines for the Institutional Animal Care and Use Committee of Chang Gung University.

### Cutaneous absorption test

The *in vitro* cutaneous absorption of resveratrol and pterostilbene was conducted using Franz diffusion cell. The excised dorsal skin of the nude mouse was mounted between the donor and receptor with the stratum corneum (SC) facing upward from the donor. The donor and receptor were filled with 20% propylene glycol (PG)/pH 7.4 buffer (0.5 ml) and 30% ethanol/pH 7.4 buffer (5.5 ml), respectively. Either the supersaturated suspension (15 mM) or the saturated solution of the compounds was placed in the donor to examine permeability. The other processes and the permeant amount extracted from the skin were the same as in our previous study (Lin et al., 2016). Both the skin deposition (nmol/mg) and flux (nmol/cm^2^/h) were determined as the permeation capability for the supersaturated suspension. The flux was calculated by a linear regression from the slope of the penetrated amount-time curves. In the case of the saturated solution, the calibrated skin accumulation (CSA) and permeability coefficient (K_*p*_) were computed using the skin deposition and flux divided by the saturated solubility (applied permeant dose) in the donor, respectively.

### *In vivo* cutaneous MRSA infection

The nude mouse back was intradermally injected with MRSA (1 × 10^7^ CFU) in PBS (150 μl). Then 20% PG/pH 7.4 buffer (control group) or pterostilbene (15 mM) with a volume of 0.2 ml was topically applied above the injected site every 24 h for 7 consecutive days. The macroscopic appearance of the skin was monitored at day 1, 3, and 7. Transepidermal water loss (TEWL, TM300, Courage and Khazaka, Köln, Germany) and the yellow-green color (b*) of the skin were analyzed from 0 to 7 days post-administration. At the end of the experiment (day 7), the mouse was sacrificed and the treated skin site was excised for histological examination and MRSA counting. The skin specimen was homogenized by MagNA Lyser (Roche, Indianapolis, IN, USA). The bacterial cell number was determined by plating serially diluted skin homogenate on TSB for 24 h. The CFU was estimated as the corresponding number of MRSA.

### Keratinocyte cytotoxicity

The possible cytotoxicity of resveratrol and pterostilbene on keratinocytes (HaCaT cells) was evaluated by MTT assay as described previously (Pan et al., 2015). Briefly, HaCaT cells were seeded in a 96-well plate with 1 × 10^4^ cells/well at 37°C for 24 h. Each well was treated with resveratrol or pterostilbene at 0.01, 0.05, and 0.125 mM for 24 h. MTT (0.5 mg/ml) was pipetted into the well and then incubated for 3 h. The ELISA reader at 550 nm was used to estimate the cell viability (%). All procedures were done in the dark.

Intracellular adenosine triphosphate (ATP) was also detected by a bioluminescence assay based on the ATP-dependent luciferin-luciferase reaction using a commercial kit as described previously (Hidalgo and Domínguez, 1998). To determine the viability by the ATP content, after 24 h of culture, 100 μl of CellTiter-Glo® reagent (Promega, Madison, WI, USA) was added to the wells. The mixture was shaken for 5 min to elicit cell lysis. The plate was incubated at room temperature for 10 min to stabilize the luminescent signal. The luminescence was detected with a luminometer (Chameleon V, Hidex, Finland).

### Human neutrophil cytotoxicity

The human neutrophils were obtained from healthy volunteers, all between 20 and 30 years of age, using a protocol approved by the Institutional Review Board at Chang Gung Memorial Hospital. The neutrophil-purification procedure was performed according to our previous study (Yang et al., 2016). The neutrophils (6 × 10^5^ cells/well) were incubated with the compounds (0.01, 0.05, and 0.125 mM) for 15 min at 37°C. Lactate dehydrogenase (LDH) leakage from the neutrophils was determined using a CytoTox 96® kit (Promega, Fitchburg, WI, USA). The cytotoxicity was calculated by LDH release in cell-free medium as a percentage of total LDH release. The complete LDH leakage was detected by 0.1% Triton X-100 exposure.

### *In vivo* cutaneous irritation

The 20% PG/pH 7.4 buffer with or without pterostilbene (15 mM) at a volume of 0.6 ml was applied daily on the nude mouse back for 7 consecutive days. The application area was 1.5 × 1.5 cm^2^. The experimental protocol was the same as with the previous work (Pan et al., 2014). After a 7-day administration, the treated skin area was monitored by macroscopic and microscopic observations. Hematoxylin and eosin (H&E) were utilized to stain skin slices for histological observation under optical microscopy (Olympus IX81, Tokyo, Japan).

### Proteomic analysis

The cultured MRSA was treated by resveratrol or pterostilbene at 2 mM for 2 h. The bacterial protein preparation protocol was the same as the method of total-protein-amount measurement. The SDS-PAGE was performed with a 5% stacking gel and a 10% separating gel followed by Coomassie blue staining. The stained bands were withdrawn and digested by trypsin at 37°C overnight. The digested proteins were acidified with 0.5% trichloroacetic acid and then loaded into an AnchorChip® 600/384. A Bruker Ultraflex® spectrometer (Bremen, Germany) was used to analyze the MALDI-TOF/TOF mass. The detailed procedures were shown in our previous study (Pan et al., 2010).

### Molecular modeling for compound-protein interaction

The crystal structure of the MRSA proteins was used for molecular docking with resveratrol and pterostilbene by Discovery Studio® (Accelrys, San Diego, CA, USA). The superimposition of the compounds with these proteins was calculated to observe the conformation and ligand-binding activity. The dock score energy was also computed after conducting the molecular docking simulation of resveratrol or pterostilbene with the proteins.

### Statistical analysis

The data presented as the mean and standard deviation (*S.D*.). The difference in the data of the different experimental groups was analyzed by the Kruskal-Wallis test. The *post-hoc* test for checking individual differences was Dunn's test. Significance was indicated as *p* < 0.05.

## Results

### Comparison of antibacterial activity between resveratrol and pterostilbene

We assessed the antibacterial activity of resveratrol and pterostilbene against a panel of pathogens representing MRSA, clinical strains of MRSA and VISA, and *P. aeruginosa*. Table 1 lists the MIC and MBC ranges for the compounds against six different species. In contrast to resveratrol, pterostilbene demonstrated potent inhibitory and biocidal activities against the Gram-positive bacteria. The MIC of pterostilbene for the drug-resistant *S. aureus* was 8~16-fold lower than resveratrol. A significant 128-fold reduction in the MBC of pterostilbene was observed when compared to resveratrol. To examine whether the compounds could suppress the growth of Gram-negative bacteria, the susceptibility of *P. aeruginosa* to the two agents was evaluated. Resveratrol and pterostilbene were basically equipotent to *P. aeruginosa* growth inhibition. The potency for killing *P. aeruginosa* could be regarded as low.

**Figure 2:**
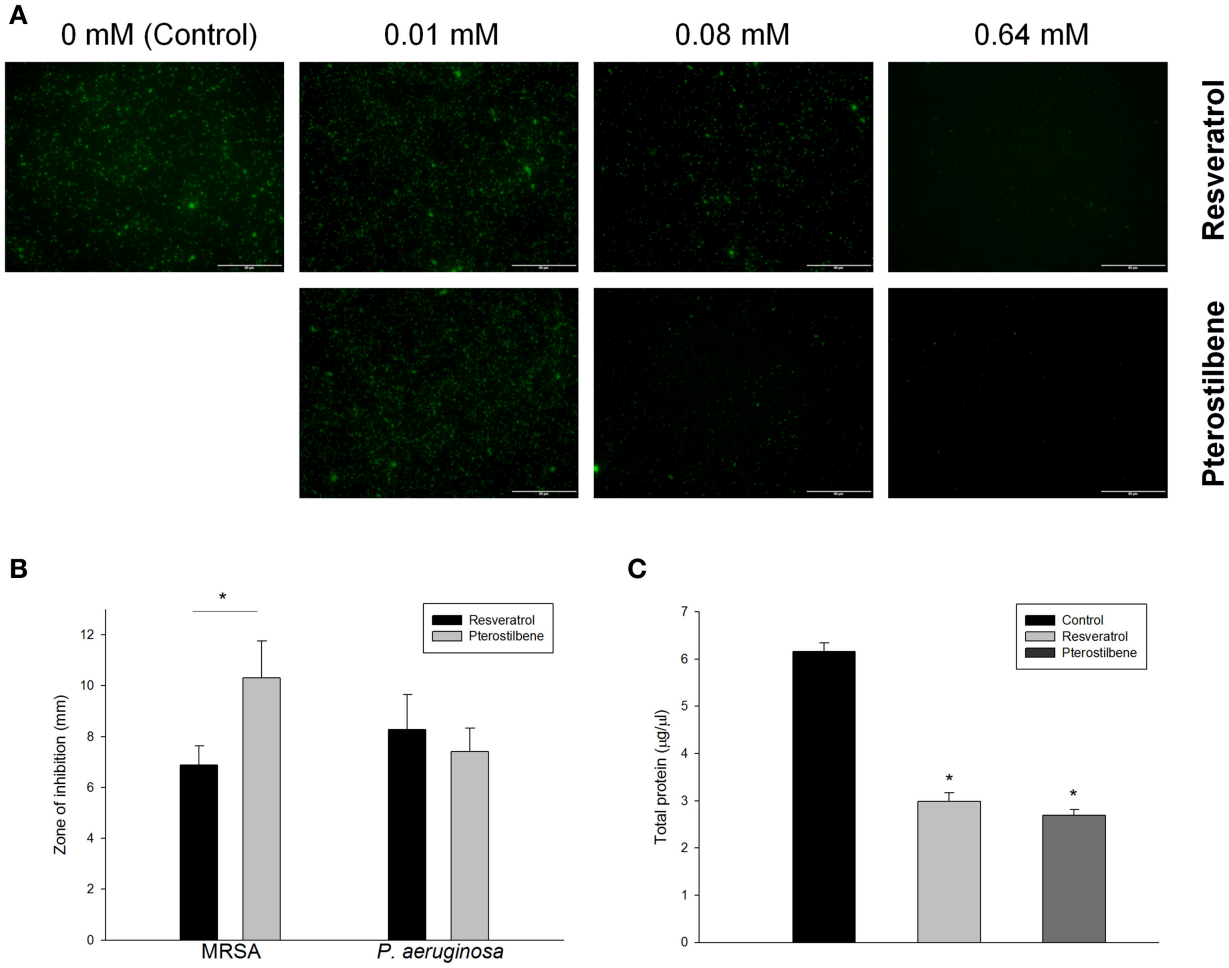
Antibacterial activity of resveratrol and pterostilbene: **(A)** the live MRSA strain viewed under confocal microscopy, **(B)** zone of inhibition measured from disk diffusion assay, and **(C)** total protein amount in MRSA. Each value represents the mean and SD (*n* = 4).

The live MRSA strain (ATCC 33591) was viewed under confocal microscopy as illustrated in Figure 2A. Resveratrol and pterostilbene did not affect MRSA viability at the concentration of 0.01 mM but significantly reduced the growth at the higher concentrations when compared with the control. Pterostilbene mediated less viable MRSA as compared to resveratrol exposure. The growth inhibition of MRSA by the natural compounds was also recognized using an agar diffusion assay. The inhibition zone with a diameter of 6.9 mm was measured for resveratrol and 10.3 mm for pterostilbene (Figure 2B). No significant difference in the inhibition zones between the two compounds was detected for *P. aeruginosa*. Protein reduction deals with the stress response in bacteria treated with antibiotics. The total protein amount in MRSA is estimated after the application of the compounds as shown in Figure 2C. Resveratrol and pterostilbene caused a 51 and 56% decrease in total protein compared to the control, respectively. The protein quantity of MRSA after resveratrol and pterostilbene treatments was comparable.

### Antibacterial activity of pterostilbene against biofilm and intracellular MRSA

**Figure 3:**
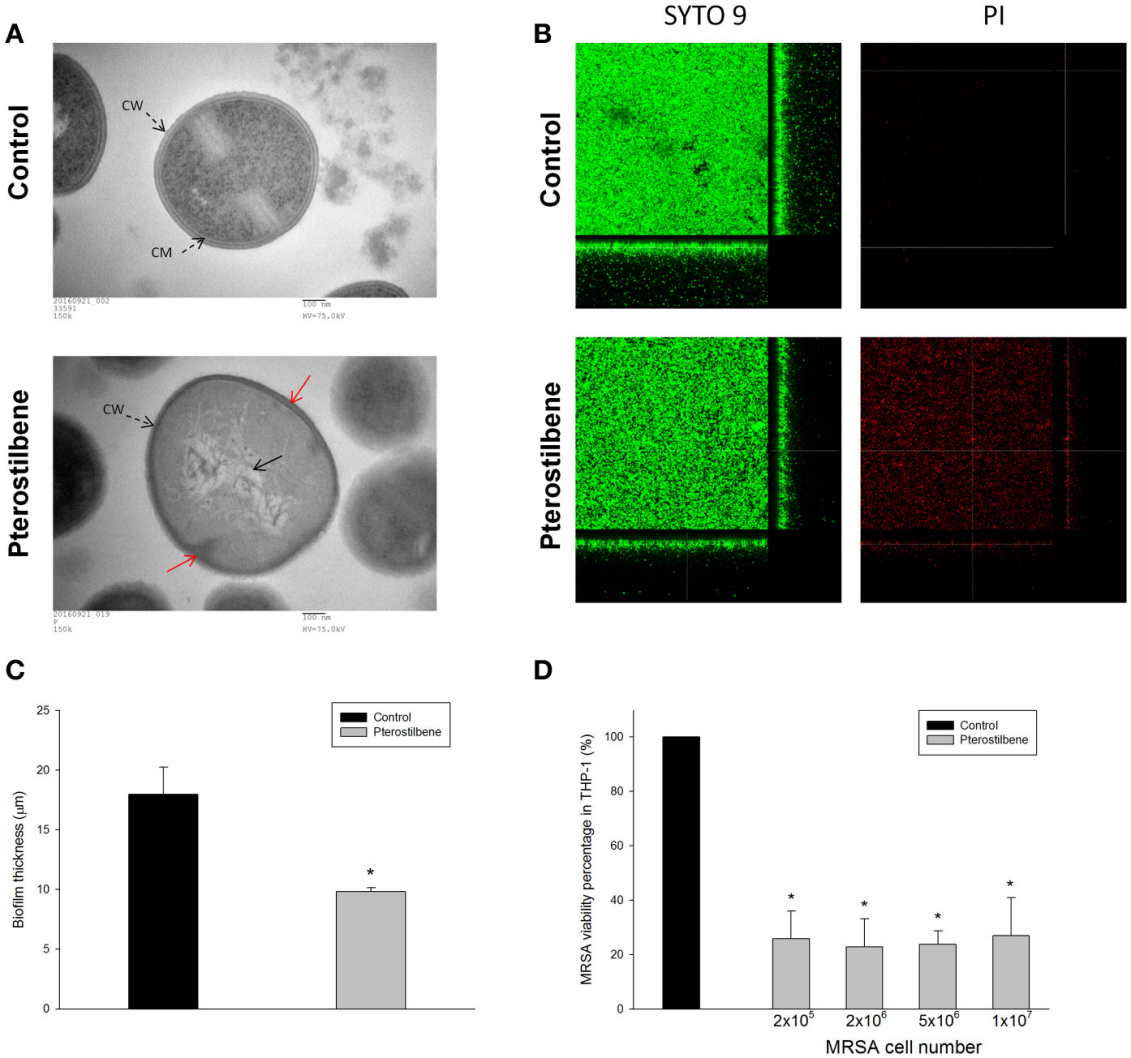
Anti-MRSA activity of pterostilbene: **(A)** Morphological changes of MRSA viewed under TEM, **(B)** the three-dimensional images of biofilm, **(C)** the corresponding biofilm thickness, and **(D)** intracellular MRSA killing in macrophages (THP-1). Each value represents the mean and S.D. (*n* = 4).

Figure 3A depicts the TEM photographs of MRSA morphology with and without pterostilbene exposure for 4 h. The intact MRSA (control) displayed a smooth and bright appearance on the cellular surface. When the bacteria were treated with pterostilbene, the cell wall/membrane perturbation was visualized (red arrow). The cytoplasm of pterostilbene-treated bacteria became amorphous and empty, suggesting the loss of cytoplasmic contents. The grainy appearance typical for intact cells was lost as a result of pterostilbene treatment. These observations may indicate that osmotic disturbance caused the MRSA damage.

Figure 3B presents the anti-biofilm effect of pterostilbene against MRSA observed by confocal microscopy. Biofilm formation is a predominant virulence factor of MRSA. We stained the viable and dead bacteria using SYTO9 and propidium iodide (PI), respectively. The viable fraction of intact biofilm (green color) showed a well-developed and dense morphology without significant bacterial death (red color). Pterostilbene was active against MRSA biofilm formation due to the decrement of biomass and thickness. As shown in Figure 3C, pterostilbene incubation reduced biofilm thickness from 18 to 10 μm. The MRSA death rate in biofilm was significantly increased by pterostilbene. MRSA is difficult to eliminate with traditional antibiotics because of the intracellular persistence. Macrophages are the major immune cells present as the first line of defense against endocytosis bacteria. We evaluated whether pterostilbene could be endocytosed by macrophages for intracellular killing of MRSA. THP-1 cells were infected by different numbers of MRSA as shown in Figure 3D. The treatment of infected THP-1 with pterostilbene resulted in a dramatic reduction of live MRSA. This treatment did not cause any cytotoxic effect on THP-1. A decrease of about 75% in intracellular MRSA survival was detected for pterostilbene treatment. There was no significant difference in the bacterial inhibition percentage among pterostilbene applications on different MRSA burdens.

### Cutaneous absorption test

Cutaneous absorption of resveratrol and pterostilbene was compared by Franz cell assembly. Both the skin deposition and penetration across the skin were estimated. The permeant absorption by the skin can be measured as skin deposition, while the cumulative amount in the receptor anticipates the delivery into the deeper skin strata or systemic circulation. The donor permeant concentration was first fixed at an equivalent dose (15 mM). This dose was higher than the saturated solubility of both compounds in a 20% PG vehicle. As summarized in Table 2, the skin deposition and flux of pterostilbene are much higher than those of resveratrol. The delivery of pterostilbene into the skin was 6-fold greater than the delivery of resveratrol. The permeants in the donor were also dosed with saturated solution to keep a constant driving force with maximum thermodynamic activity. Table 2 demonstrates the CSA and K_*p*_ in nude mouse skin. With an equivalent dose, pterostilbene exhibited a higher skin delivery as compared to resveratrol. We showed a 5-fold rise in the deposition compared to resveratrol. To rate the possible anti-MRSA activity of the compounds after topical application, the therapeutic index (TI) was calculated based on multiplying the skin deposition and the anti-MRSA inhibition zone as presented in Figure 2B. As shown in Table 2, the TI of pterostilbene is greater than that of resveratrol in both conditions of equivalent and saturated doses. Pterostilbene gave an 8~9-fold greater TI than resveratrol, with the expectation of an effective antimicrobial capability of topically applied pterostilbene.

**Table 2:** Nude mouse skin permeation parameters of resveratrol and pterostilbene after in vitro percutaneous absorption from 20% PG/pH 7.4 buffer suspension at a determined concentration (15 mM) and at a saturated concentration.

**Compound**	**Suspension (mM)**			**Saturated solution**		
	**Skin deposition (nmol/mg)**	**Flux (nmol/cm^2^/h)**	**TI^a^**	**CSA^b^ (nmol/mg/solubility)**	**K*_p_*^c^(cm/h × 10^−3^)**	**TI^a^**
Resveratrol	1.71 ± 0.62	5.98 ± 1.20	11.78	0.24 ± 0.05	2.70 ± 2.64	1.65
Pterostilbene	10.40 ± 1.03	41.38 ± 1.14	107.33	1.29 ± 0.21	13.00 ± 1.11	13.31

a *TI, therapeutic index = skin deposition or CSA x antibacterial inhibition zone*.

b *CSA, calibrated skin accumulation = cumulative amount in the skin/saturated solubility*.

c *K_p_, permeability coefficient = flux/saturated solubility*.

*The data represent the mean ± S.D. (n = 4)*.

### *In vivo* cutaneous MRSA infection

**Figure 4:**
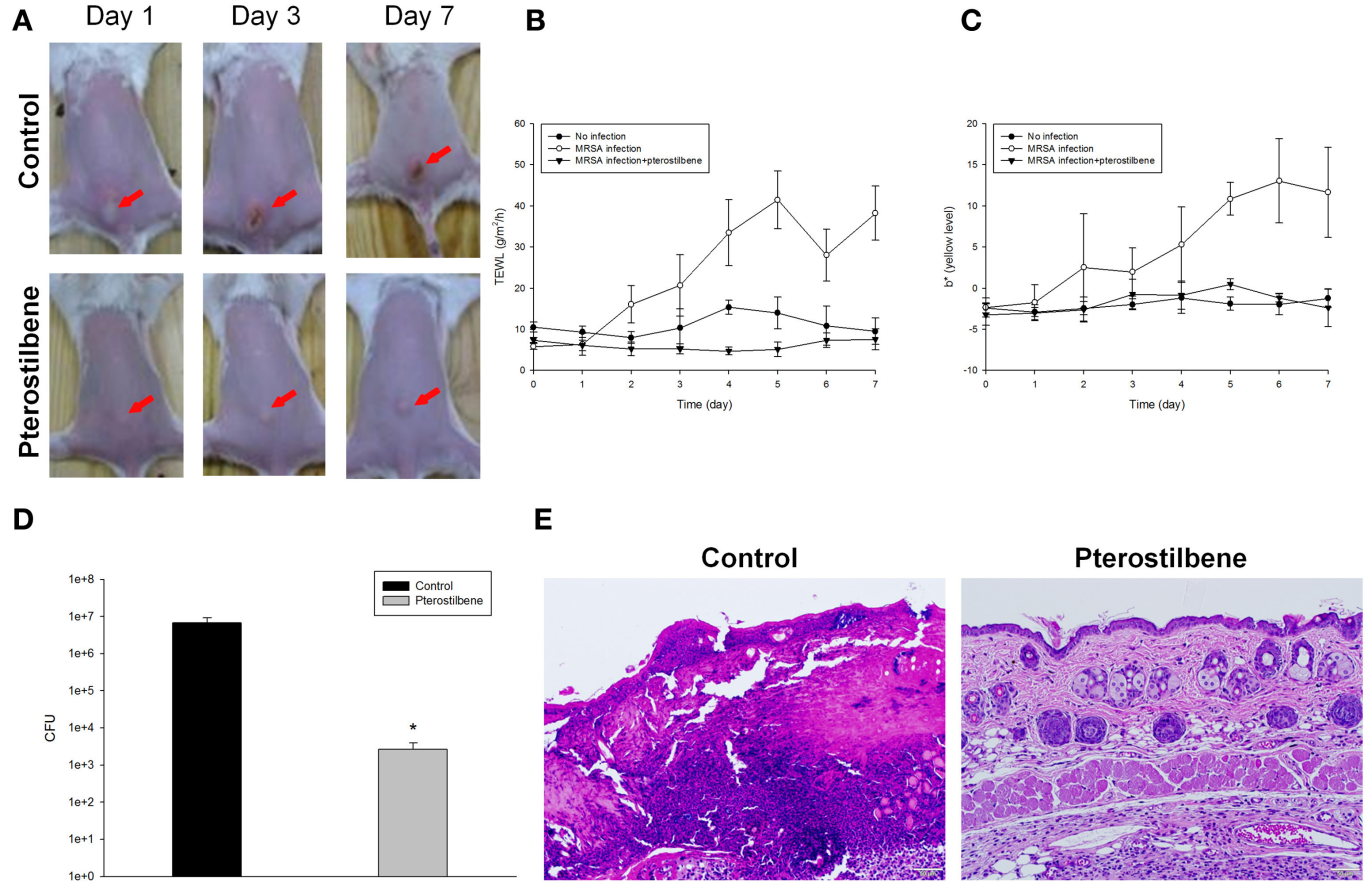
*In vivo* topical application of pterostilbene against MRSA: **(A)** The skin surface of mice after treatment of MRSA at day 1, 3, and 7; **(B)** Transepidermal water loss (TEWL) of mice skin treated with MRSA; **(C)** Yellow/blue color (b*) of mice skin treated with MRSA; **(D)** Survival of MRSA in mice skin treated with MRSA. Seven days after infection, skin lesions were cut, homogenized, and bacterial count was determined by CFU assay, and **(E)** Histological observation of mice skin biopsy after treatment of MRSA. On day 7, biopsy specimens were taken immediately after the termination of the experiment and stained with hematoxylin and eosin (H&E). Scale bar = 50 μm. Each value represents the mean and S.D. (*n* = 6).

To further determine the therapeutic efficiency of pterostilbene, the nude mouse was challenged with MRSA followed by topical delivery of vehicle control or pterostilbene. The macroscopic observation in Figure 4A reveals the skin infection in the control group, which can be seen by the presence of abscess at the injection area (red arrow) after 1 day of injection. The abscess worsened following the increase of incubation time. Application of pterostilbene significantly restrained the size of eschar. There is only a phyma (red arrow) in the injection site after pterostilbene exposure. TEWL was monitored daily to evaluate the skin-barrier function as depicted in Figure 4B. Pterostilbene significantly ameliorated the disrupted skin barrier produced by MRSA invasion. The TEWL for pterostilbene application approximated the non-treatment control. The abscess-generated pus revealed a yellow-brown appearance. The yellow-brown color can be quantified by the b* axis of colorimetry. As shown in Figure 4C, the yellow-brown level of the injection site in the non-treated control increased following the increase of incubation time. This yellow-brown color could be inhibited by pterostilbene.

Seven days after infection, the mouse was sacrificed and the MRSA burden in the skin was determined. Pterostilbene was effective at inhibiting MRSA growth, resulting in about a 3-log CFU lessening compared to the control (Figure 4D). We next examined the skin architecture by H&E staining. As seen in Figure 4E, the skin section of MRSA infection (control) reveals total damage to the epidermis, degenerated dermis, and inflammatory cell infiltration. The MRSA burden was mixed with the immune cells in the dermis and subcutis, suggesting a deep inflammation. The infected skin exposed to pterostilbene showed nearly normal features. The immune cell infiltration was largely restricted in the pterostilbene-treated skin.

### Effect of resveratrol and pterostilbene on cytotoxicity

**Figure 5:**
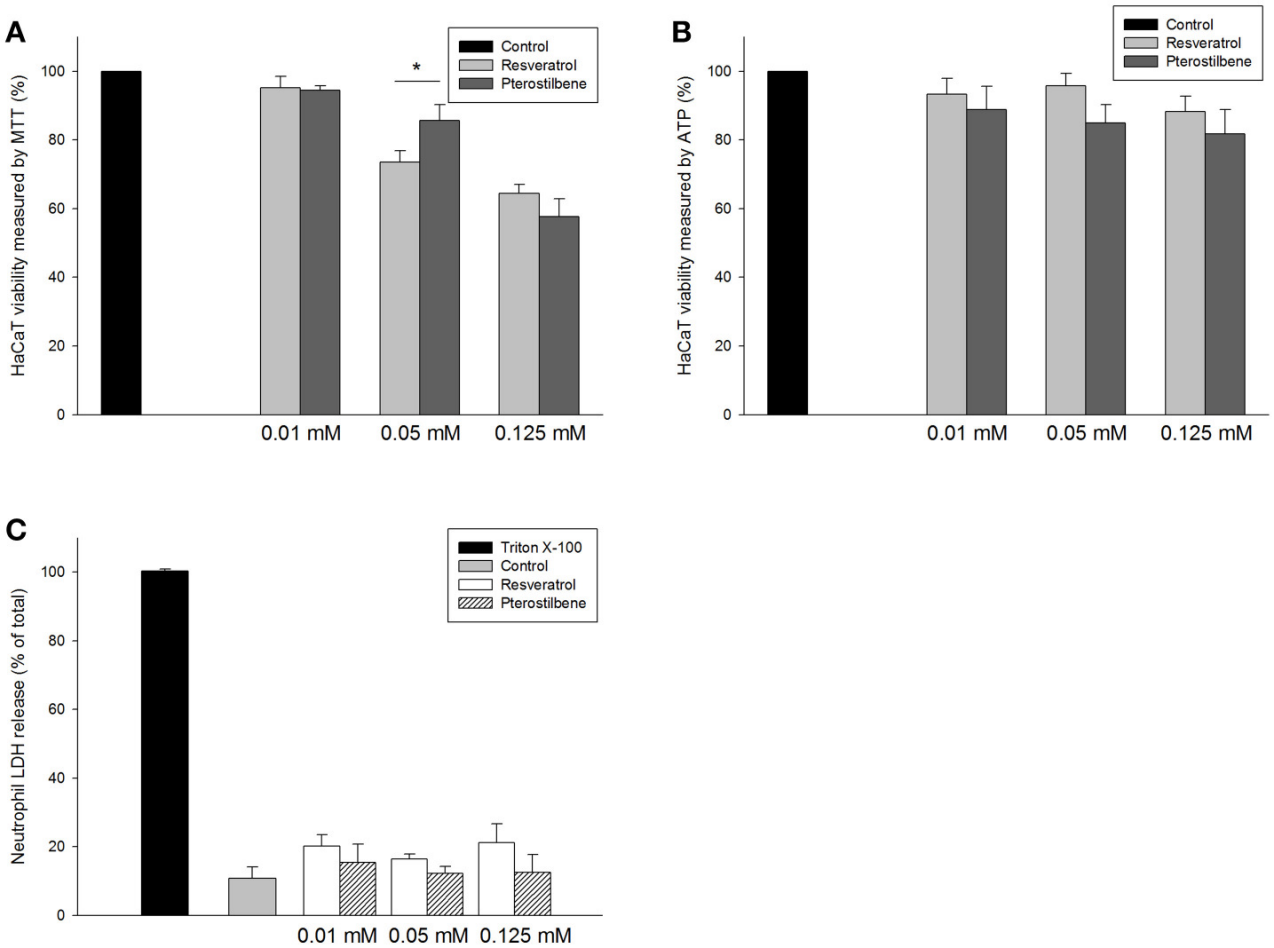
*In vitro* cytotoxicity of resveratrol and pterostilbene to: **(A)** HaCaT keratinocyte cell viability measured by MTT assay; **(B)** HaCaT keratinocyte cell viability measured by ATP assay; and **(C)** human neutrophils determined by LDH release. Each value represents the mean ± S.D. (*n* = 4). ^*^p < 0.05.

The application of the antibacterial drugs can be limited because of the cytotoxic effect on mammalian cells. The prerequisite of antibacterial agent development is the assurance of safety. As shown in Figure 5A, resveratrol and pterostilbene at 0.01 mM were found to be non-toxic to keratinocytes, whereas a mild cytotoxicity at the higher concentrations was detected. A higher level of cell viability was observed for pterostilbene (86%) over resveratrol (74%) at the dose of 0.05 mM. We also employed intracellular ATP analysis for evaluating HaCaT viability as shown in Figure 5B. The viability could be maintained to >80% after treatment of resveratrol or pterostilbene at the concentration range of 0.01~0.125 mM. Neutrophils were also examined for viability since there is neutrophil-rich infiltrate in the MRSA-infected skin wounds. The neutrophil cytotoxicity can be determined by LDH release associated with membrane damage. LDH assay indicates that resveratrol and pterostilbene at the concentrations of 0.01~0.125 mM can generally maintain neutrophil viability to >80% as shown in Figure 5C. There was no significant difference of LDH leakage between the control and the compound-treated groups.

### *In vivo* cutaneous irritation

**Figure 6:**
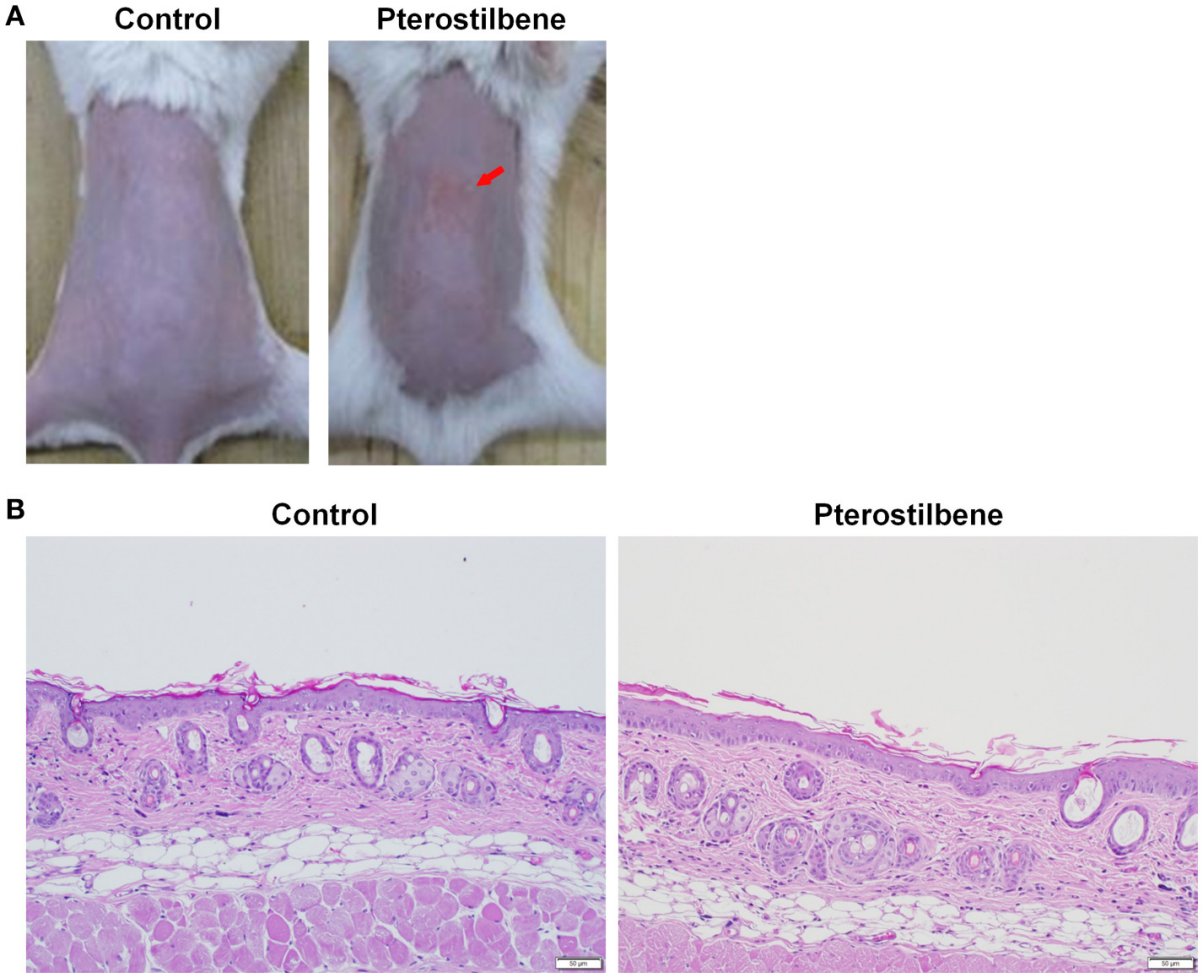
Skin tolerance examination of mouse skin a 7-day treatment of topically applied pterostilbene: **(A)** Macroscopic appearance and **(B)** skin specimen stained with hematoxylin and eosin (H&E).

The safety of topically applied pterostilbene was elucidated using a skin irritation test on nude mice. Figure 6A shows the macroscopic appearance of the dorsal skin after pterostilbene administration. A slight erythema (red arrow) visualized at the pterostilbene-treated site could be categorized as very mild. The possible irritation of the skin was further checked by microscopic examination as illustrated in Figure 6B. The vehicle control displayed an intact morphology without damage. The biopsied skin specimen after pterostilbene exposure showed a comparative structure vs. the control skin, suggesting a preferred biocompatibility to the skin.

### Proteomic analysis

**Figure 7:**
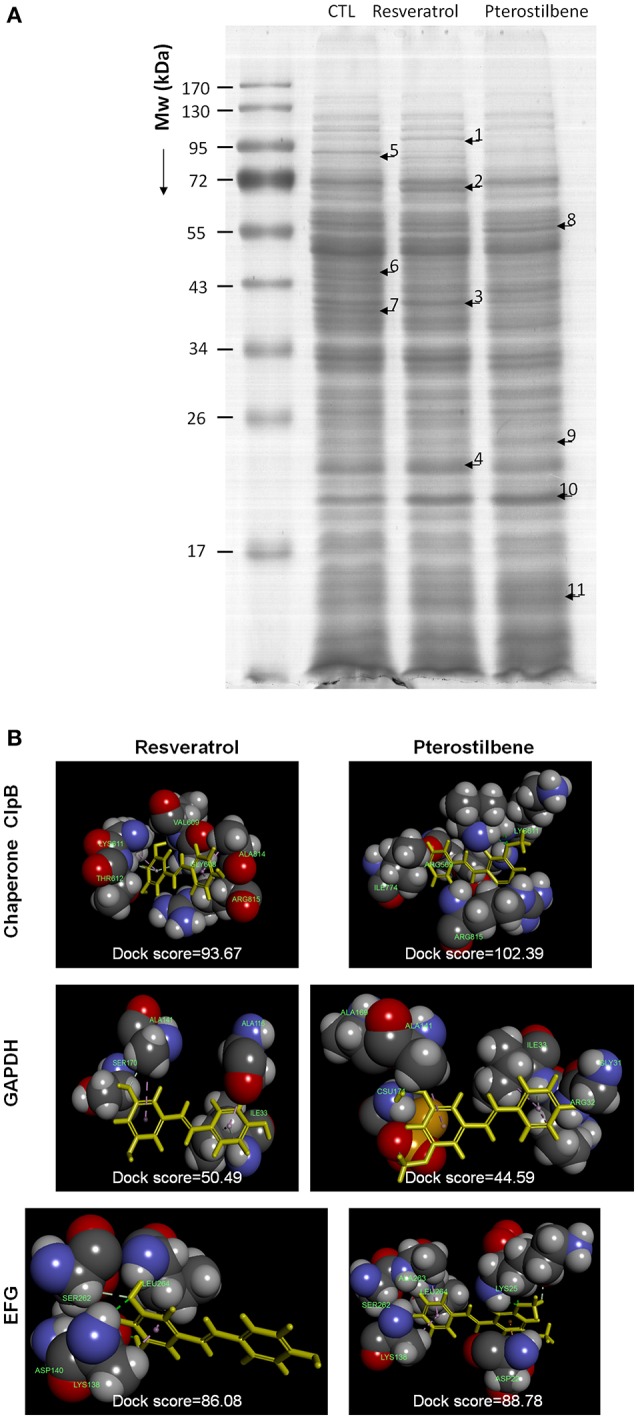
The effect of resveratrol and pterostilbene on MRSA proteins: **(A)** The protein change of MRSA after treatment with resveratrol and pterostilbene analyzed by SDS-PAGE and MALDI-TOF/TOF mass and **(B)** Superimposition of the computed poses for resveratrol/pterostilbene and proteins. The molecular docking was calculated by Discovery Studio® (Accelrys, San Diego, CA, USA).

The 2D protein gel electrophoresis combined with MALDI-TOF/TOF mass was employed to analyze the protein profiles of MRSA after treatment with resveratrol and pterostilbene to elucidate the antibacterial mechanisms. Figure 7A shows the results of SDS-PAGE. The protein bands of resveratrol- and pterostilbene-treated MRSA were quite different from those of non-treated bacteria. Eleven protein bands differentially expressed in MRSA after compound exposure are labeled in Figure 7A. The significant change in the proteins elicited by resveratrol and pterostilbene was determined by mass spectra as demonstrated in Table 3. The band numbers in Figure 7 refer to those in Table 3. With respect to resveratrol, seven proteins were upregulated. On the other hand, pterostilbene caused the increased expression of five proteins. Chaperone proteins and glyceraldehyde-3-phosphate dehydrogenase (GAPDH) were upregulated in resveratrol-treated MRSA but downregulated in the pterostilbene-treated group. The expression of 30S and 50S ribosomal proteins was increased after compound treatment, with pterostilbene showing greater upregulation. A similar trend was observed for alkaline shock protein (ASP).

**Table 3:** Differentially expressed proteins follow resveratrol and pterostilbene treatments.

**Band No**.	**Protein**	**Accession no**.	**Mw (Da)**	**Matched-peptides**	**Sequence coverage % (SCORE)**	**Ratios to control^a^**			**Biological function**
						**Resveratrol**	**Pterostilbene**	***p*-value^b^**	
1	Chaperone protein ClpB (CLPB)	Q6GAV1	87,165	27	45% (191)	3.85 ± 0.04	−1.26 ± 0.02	0.038	Part of a stress-induced multi-chaperone system, it is involved in the recovery of the cell from heat-induced damage, in cooperation with DnaK, DnaJ, and GrpE.
2	Chaperone protein DnaK (DNAK)	P64408	66,321	18	43% (123)	5.41 ± 0.07	−1.50 ± 0.04	0.041	Acts as a chaperone.
3	Glyceraldehyde-3-phosphate dehydrogenase (GAPDH)	P0A038	36,382	12	48% (117)	1.50 ± 0.02	−3.82 ± 0.06	0.028	Catalyzes the oxidative phosphorylation of glyceraldehyde 3-phosphate (G3P) to 1,3-bisphosphoglycerate (BPG) using the cofactor NAD.
4	50S ribosomal protein L6 (RL6)	Q7A084	19,802	10	62% (96)	1.62 ± 0.11	2.27 ± 0.01	0.036	This protein binds to the 23S rRNA, and is important in its secondary structure. It is located near the subunit interface in the base of the L7/L12 stalk, and near the tRNA binding site of the peptidyltransferase center.
5	Elongation factor G (EFG)	P68791	76,877	29	45% (212)	−3.19 ± 0.07	−1.47 ± 0.13	0.025	Catalyzes the GTP-dependent ribosomal translocation step during translation elongation.
6	Arginine deiminase (ARCA)	Q8NUK7	47,069	20	46% (145)	−2.57 ± 0.03	−1.50 ± 0.05	0.036	L-arginine + H_2_O = L-citrulline + NH_3_.
6	Glucose-6-phosphate isomerase (G6PI)	Q8NXF1	49,849	14	48% (93)	−2.26 ± 0.05	−1.42 ± 0.01	0.048	D-glucose 6-phosphate = D-fructose 6-phosphate.
7	Citrate synthase 2 (CISY2)	P39120	40,614	10	31% (92)	−3.37 ± 0.02	−2.17 ± 0.02	0.013	Might regulate the synthesis and function of enzymes involved in later enzymatic steps of Krebs cycle. Loss in activity results in sporulation defect.
8	Phosphoenolpyruvate carboxykinase [ATP] (PCKA)	P0C1S4	58,803	19	36% (102)	1.52 ± 0.04	2.83 ± 0.01	0.032	Involved in the gluconeogenesis. Catalyzes the conversion of oxaloacetate (OAA) to phosphoenolpyruvate (PEP) through direct phosphoryl transfer between the nucleoside triphosphate and OAA.
9	30S ribosomal protein S4 (RS4)	P66564	23,027	21	61% (159)	−1.40 ± 0.05	2.69 ± 0.01	0.018	One of the primary rRNA binding proteins, it binds directly to 16S rRNA where it nucleates assembly of the body of the 30S subunit.
10	Alkaline shock protein 23 (ASP)	P0A0P7	19,210	11	68% (124)	1.59 ± 0.01	2.93 ± 0.01	0.021	May play a key role in alkaline pH tolerance.
11	30S ribosomal protein S13 (RS13)	P66389	10,343	10	89% (92)	1.59 ± 0.01	2.03 ± 0.05	0.010	May function as a redox-sensitive chaperone and as a sensor for oxidative stress.

a *Ratios to control indicated the fold changes in protein volume between resveratrol-, pterostilbene-treated samples vs. control samples, respectively. The higher ratios (>1.0) mean the proteins whose expression levels were increased upon treatments of compounds, while lower ratios (<−1.0) indicate the proteins were downregulated under the exposure to compounds*.

b *p-values were generated by analyzing the gel images using Prodigy SameSpots™ software. Differences were considered significant at p < 0.05 after the comparison of the ratios to control between resveratrol and pterostilbene groups*.

The possible interaction between the compounds and the selected proteins was examined by a computational study of best docking poses as shown in Figure 7B. Both compounds had exhibited the ligand-binding activity to chaperone ClpB but different conformations. The dock score was compared after conducting the molecular docking simulation of the compounds with the proteins as demonstrated in Figure 7B. A greater dock score indicates a stable system for a likely binding interaction. We found a stronger binding of chaperone to pterostilbene (dock score = 102) than to resveratrol (dock score = 94). Contrary to this result, a comparable interaction of GAPDH to resveratrol (dock score = 50) and pterostilbene (dock score = 45) was observed. The conformation orientation of resveratrol and pterostilbene to elongation factor G (EFG) was similar, with pterostilbene possessing more amino acid interactions. There was no interaction between ribosomes and the compounds after docking the conformation.

## Discussion

The drug resistance of planktonic, biofilm, and intracellular bacteria highlights the need to develop new antibacterial agents. Here we report the discovery that pterostilbene, a natural product, demonstrated antimicrobial activity against MRSA and VISA. Pterostilbene offered superior antibacterial potency compared to resveratrol. The proteomic profiles suggested that the mode of action of pterostilbene was different from that of resveratrol. Pterostilbene was shown to induce cell membrane disruption. It was also observed that pterostilbene reached a therapeutic level to eradicate MRSA in biofilm and the infected host cells. The *in vitro* cytotoxicity of mammalian cells and an *in vivo* skin irritation test had confirmed the preliminary safety of this compound.

Most MRSA found in clinical settings is multidrug resistant (Cihalova et al., 2015). After its introduction in 1958, vancomycin served as the last line of defense for anti-MRSA therapy. However, the emergence of vancomycin-resistant bacteria was reported in the early 2000s (Chang et al., 2016). The findings in the *in vitro* antibacterial test intensified the potential of pterostilbene for the treatment of infection caused by *S. aureus* bacteria that are resistant to methicillin and vancomycin. The antibacterial activity of pterostilbene against *P. aeruginosa* was not prominent. The previous study (Paulo et al., 2010) indicates a lesser antimicrobial effect of resveratrol against Gram-negative bacteria than Gram-positive bacteria. This could be because Gram-negative bacteria are structurally and chemically complex. The lack of inhibitory effect on Gram-negative bacteria led to the prediction that pterostilbene would be a drug candidate for specifically treating diseases involving staphylococci.

The phenotypic drug resistance of *S. aureus* has been attributed to multiple factors, including the biofilm activity as a permeation barrier and the intracellular retention in immune cells (Nair et al., 2016). MRSA is known to form biofilm composed of an extracellular polymeric substance matrix, which is tough to combat with conventional antibiotics and host defense. Biofilm fabrication is an essential factor contributing to *S. aureus* infection in the skin (Archer et al., 2011). An ideal anti-MRSA agent should be able to penetrate and destroy the biofilm matrix. Our results indicated that pterostilbene had the activity to eradicate biofilm. The reduction of biofilm formation by pterostilbene was related to the MRSA killing inside the biofilm according to the confocal imaging. The dead bacteria were visualized throughout the full thickness of the biofilm. The biofilm might not be a main barrier for pterostilbene delivery. Pterostilbene was inferred to stick to the biofilm surface, penetrate the matrix, and then distribute all over the depth of the biofilm. Most antibacterial drugs have poor cellular permeation, resulting in restricted intracellular distribution and unsatisfactory antibacterial activity. For instance, vancomycin is unable to show a biocidal effect on MRSA residing in macrophages (Pumerantz et al., 2011). Antibiotics such as aminoglycosides and β-lactams possess limited cellular penetration owing to low lipophilicity (Mu et al., 2016). The lipophilicity of pterostilbene (log *P* = 3.54) is greater than that of resveratrol (log *P* = 3.09). The macrophages might tend to engulf pterostilbene, making this compound a suitable agent to kill MRSA inside the immune cells.

The cutaneous absorption of resveratrol and pterostilbene had demonstrated a similar trend either at the equivalent dose or at the dose of saturated solubility, with pterostilbene showing higher skin delivery than resveratrol. A topically applied drug in aqueous vehicle first partitions and accumulates into the SC, which is rich in lipids. The higher lipophilicity of pterostilbene contributed to the facile deposition in the skin. Previous studies (Lin et al., 2012, 2016) showed that the use of methoxylation was an efficient way to enhance permeant lipophilicity for increased entry into the SC lipid bilayers. Ceramides are the predominant lipids in the SC. The hydrogen bond donor number of the permeant has been recognized as impacting interaction with the head groups of ceramides (Patel et al., 2002; Liu et al., 2011). The hydrogen bond donor number of resveratrol and pterostilbene is 3 and 1, respectively, based on the measurement by Discovery Studio®. Fewer hydrogen bond numbers in the permeants may accelerate the diffusion because of the increased permeant transport into the non-polar region of the SC. The greater absorption of pterostilbene than resveratrol was also approved in the case of oral delivery. Pterostilbene was reported to have an oral bioavailability of 80% compared to 20% for resveratrol due to the presence of two methoxyl moieties of pterostilbene (Kapetanovic et al., 2011). The assessment of TI based on *in vitro* results is an easy method with the use of the preliminary measurement of topically applied agents. A high skin deposition and antibacterial activity of pterostilbene had led to an impressive TI for therapeutic intervention. Topically applied pterostilbene created a cutaneous reservoir in the skin that could prolong the residence time to eliminate pathogenic microbes.

Approximately 75% of community-associated MRSA infection occurs in the skin (Kurosu et al., 2013). The bacterial abscess caused by MRSA is difficult to treat using conventional antibiotic therapy due to the biofilm-like nature (Han et al., 2009). We appraised the *in vivo* anti-MRSA efficacy of pterostilbene in the nude mouse abscess model, which resembled localized cutaneous infection. Topically applied pterostilbene facilely diffused into the skin and abscess to form a reservoir. The depot of pterostilbene facilitated the efficient delivery to the targeted infection region. The MRSA burden, skin barrier function, and inflammation were significantly improved by pterostilbene.

A number of antimicrobial formulations can cause local reactions such as erythema, rash, burning sensation, and tenderness when applied topically (Verma and Pathak, 2012). We showed *in vitro* that pterostilbene had tolerable levels of toxicity toward cultured keratinocytes. The *in vivo* safety evaluation through topical administration exhibited minimal adverse events. MRSA-infected skin is often accompanied by neutrophil and macrophage migration and infiltration for host defense (Martinez et al., 2009). Pterostilbene revealed limited toxicity against neutrophils for maintaining the host's defense ability to constrain bacterial growth. A recent clinical trial reports that oral pterostilbene at doses of 100~250 mg did not produce any significantly adverse effects (Riche et al., 2013), suggesting the safety of pterostilbene administration in humans.

The generation of oxidative stress is one of the important bacterial killing mechanisms. The cellular membrane oxidation induced by resveratrol for bacterial lethality is proposed (Subramanian et al., 2014). The previous study (Li et al., 2014) suggested that pterostilbene altered the gene expression that functions in oxidative stress in *C. albicans*. Pterostilbene exposure to cancer cells has been shown to increase reactive oxidative species (ROS) production (Mannal et al., 2010). Thus, pterostilbene may exhibit the oxidative activity needed to kill MRSA bacteria. Recently, Liu et al. (2016) reported that *S. aureus* lethality induced by ciprofloxacin and daptomycin could be antagonized by resveratrol. This interference could be due to resveratrol's strong antioxidant effect. The limited anti-MRSA activity of resveratrol shown in the present study may also relate to this offset effect. We demonstrated through TEM that pterostilbene triggered bacterial membrane perturbation, resulting in increased cell permeability. The PI staining inside the dead MRSA in the biofilm study had verified the compromised membrane integrity after pterostilbene exposure. The membrane disintegration tends to leach out the macromolecules such as DNA, RNA, and proteins. This led to the reduction of the total protein amount in MRSA treated with pterostilbene.

Although pterostilbene disrupted the membrane integrity, this effect could be considered as mild, based on the bacterial morphology. The anti-MRSA activity of pterostilbene was not only mediated by membrane damage but also by the protein expression change. The antibacterial agents possibly interact with pathogens in different locations such as the wall, membrane, and cytoplasm. We had employed a proteomic approach to characterize the functional proteins of MRSA with resveratrol and pterostilbene treatments. Both compounds revealed quite different protein expressions in MRSA. Chaperones and GAPDH were upregulated by resveratrol but downregulated by pterostilbene. Chaperones are components of the *S. aureus* wall stress regulon. There is a link between chaperones and antibiotic resistance. Chaperone proteins in *S. aureus* contribute to the adaptation to antibiotics (Frees et al., 2014). Overproduced chaperones aid the survival of *S. aureus* in environmental stress. Resveratrol might be the source of stress to activate the chaperone regulon for an adaptive response. Disruption of chaperones leads to the dramatic decrease of MRSA resistance to antibiotics (Jousselin et al., 2012). Pterostilbene significantly decreased chaperone expression in MRSA, suggesting that this agent inhibited the action of chaperones and sustained the bacteria in a state of high stress for killing the bacteria. This could lead to the greater biocidal activity of pterostilbene compared to resveratrol. GAPDH is important during *S. aureus* infection and is necessary for virulence and pathogenesis (Purves et al., 2010). GAPDH induced by oxidative stress can protect *S. aureus* against oxidative damage. This resistance against ROS is the key to survival (Weber et al., 2004). The pterostilbene-elicited GAPDH inactivation could be one mechanism for MRSA growth arrest. The antibacterial mechanisms of pterostilbene are quite different from those of resveratrol with regard to MRSA susceptibility and resistance.

Ribosomes are the resistance proteins that work against antibiotics (Costerton et al., 1999). They play a critical role in substrate stability during protein synthesis. 30S ribosome S13 and 50S ribosome L6 were upregulated by both compounds for a resistant response. The ribosome expression was higher for pterostilbene than for resveratrol. The bacteria develop two main strategies to cope with stress, namely chaperones and proteolysis. The chaperone-protease complex contributes to the quality control of Gram-positive bacteria proteins, which is essential for survival when encountering stress (Molière and Turgay, 2009). The protease is responsible for *S. aureus* ribosome cleavage (Wall et al., 2015). The chaperone overexpression by resveratrol led to the chaperone-mediated proteolysis for ribosome inactivation. This might result in the lower expression of ribosomes by resveratrol than by pterostilbene, which downregulated chaperones. Resveratrol even reduced 30S ribosome S4 expression, while pterostilbene promoted the manifestation of this protein.

Chaperones and ribosomes are possible targets for some antibiotics such as peptides, macrolides, oxazolidinones, and tetracyclines to kill the bacteria (Poehlsgaard and Douthwaite, 2005; Frees et al., 2014). In order to understand the possible interaction between the proteins and antibacterial agents, the molecular docking simulation of the compounds to the selected proteins was computed. A higher dock score demonstrates a stable system for binding interaction. Docking is a method that predicts the preferred orientation of one molecule to another molecule when bound to each other for forming a stable complex. It can be used to anticipate the strength of association or binding affinity between two molecules (Kitchen et al., 2004). Pterostilbene provided a stronger interaction with chaperone ClpB compared to resveratrol, indicating a probable target of chaperones for pterostilbene. A contrary result was shown in the case of GAPDH. There was no binding activity between the 30S and 50S ribosomes and the compounds, indicating that resveratrol and pterostilbene did not directly interact with ribosomes to evoke a bactericidal effect. The enhanced or suppressed ribosome expression by the compounds could be due to the regulation by DNA and other proteins such as chaperones and proteases.

Ribosomal protection protein (RPP) is derived from the EFG subfamily. The ribosomal protection mediated by RPP is important for drug-resistant bacteria against stress and antibiotics (Kobayashi et al., 2007). EFG enhances the translocation step in the protein synthesis of bacteria (Savelsbergh et al., 2009). The EFG downregulation by resveratrol and pterostilbene could result in the decrease of the total protein amount as shown in our results because of the synthetic disturbance. Borg et al. (2015) demonstrated that antibiotic fusidic acid is a ribosomal peptide elongation inhibitor that targets EFG to block protein synthesis. Resveratrol may be a strong elongation inhibitor since this molecule could downregulate EFG by 3.2-fold. The docking energy of EGF to resveratrol was comparable with that of pterostilbene, although pterostilbene reduced EFG expression by only 1.5-fold. The same as heat shock proteins, ASP is a stress-induced biomarker for *S. aureus* to adapt to the environment (Kuroda et al., 1995). Pterostilbene was more potent in upregulating ASP than resveratrol. This suggests that MRSA should produce more ASP for defending harsher intervention such as pterostilbene treatment. However, no docking was found between ASP and either compound.

The above data indicate that resveratrol and pterostilbene killed MRSA by disintegrating the cell surface and by changing the proteins, thereby making the bacteria susceptible to stress. The direct interaction of the compounds with the microbial membrane and key proteins could inhibit pathogen growth or cause cell death. We demonstrated that resveratrol and pterostilbene showed different mechanisms for anti-MRSA activity. The different targets might be involved in the mechanisms of the action of resveratrol and pterostilbene. It should be cautious to use proteomics and molecular docking for elucidating the anti-MRSA pathways since these techniques cannot fully explore the mechanisms or targets of the antibacterial agents. It is only a preliminary prediction of the possible targets. Further investigation is needed to explore the underlying and detailed mechanisms of action. Our aim is to offer an initial direction for the future study. More biochemical assays and genetic approaches are needed to confirm the anti-MRSA hypothesis for resveratrol and pterostilbene raised in this study.

## Conclusions

We attempted to examine the possible application of pterostilbene for therapeutic intervention of drug-resistant bacteria. Resveratrol and pterostilbene exhibited antibacterial activity without exerting unacceptable cytotoxicity on mammalian cells. Pterostilbene was more efficient for bacterial eradication than resveratrol. Pterostilbene was able to reduce biofilm formation and kill the MRSA in immune cells by virtue of its strong antibacterial activity and facile delivery across biomembranes. This natural agent also showed a 6-fold higher cutaneous absorption compared to resveratrol. Pterostilbene had unique mechanisms of action different from resveratrol according to proteomic assay. Bacterial membrane disruption and the change of stress-induced protein profiles were the biocidal mechanisms of pterostilbene. This compound is of interest as a feasible antimicrobial candidate for use against MRSA infection in skin tissues.

## Author contributions

Conceived and designed the experiments: FY and JF. Performed the experiments: SY, CT, and YW. Analyzed the data: PW. Contributed reagents/materials/analysis tools: PL. Wrote the paper: SY, FY, and JF.

### Conflict of interest statement

The authors declare that the research was conducted in the absence of any commercial or financial relationships that could be construed as a potential conflict of interest. The reviewer TC and handling Editor declared their shared affiliation, and the handling Editor states that the process nevertheless met the standards of a fair and objective review.
